# Colorectal polyp model established by transplacental BMP4 RNAi

**DOI:** 10.3892/mmr.2014.2216

**Published:** 2014-05-07

**Authors:** XIN JIN, ZHONGMEI CHEN, LI XIANG, QING LUO, ZHENGHUA GUO, XIONGHUI DING, XIANQING JIN

**Affiliations:** 1 Ministry of Education Key Laboratory of Child Development and Disorders, Children’s Hospital of Chongqing Medical University, Chongqing 400014, P.R. China; 2 Key Laboratory of Pediatrics in Chongqing, Children’s Hospital of Chongqing Medical University, Chongqing 400014, P.R. China; 3 General Hospital of Chongqing Iron and Steel Group, Chongqing 400081, P.R. China; 4 Department of Neonatal Gastrointestinal Surgery, Children’s Hospital of Chongqing Medical University, Chongqing 400014, P.R. China

**Keywords:** transplacental RNA interference, BMP4, mouse model, colorectal polyps

## Abstract

Previous studies have shown that disruption of the bone morphogenetic protein (BMP) signaling pathway is an important cause of intestinal cancer in human and animal models. Thus, the purpose of this study was to construct a Balb/C model of colorectal polyps. Pregnant mice at 9.5 days gestation were injected via the tail vein with the pSES-Si BMP4 plasmid bearing a fluorochrome (DsRed) reporter, in order to silence the *BMP4* gene in the first generation (F1); this group of mice was named the pSES-BMP4 group Intestinal fluorescence was detected at 1-, 4- and 8-week-old F1 mice, and reverse transcription-polymerase chain reaction (RT-PCR) and western-blotting assays were used to determine changes in the expression of BMP4. A dissecting microscope and hematoxylin and eosin (H&E) staining were used to observe the cell morphology and appearance of the polyps. DsRed fluorescence was observed in the intestines of 1-week-old F1 mice of the pSES-BMP4 group. BMP4 expression at the mRNA and protein level was reduced in 1-, 4- and 8-week-old F1 mice (P<0.05). However, the level of *Smad4* mRNA was only reduced in 8-week-old F1 mice (P<0.05). Multiple hyperplasic polyps emerged in the colon and rectum of the intestines of 4-week-old F1 mice in the pSES-BMP4 group. The size of colorectal polyps increased at 8 weeks, when vessels and polyp pedicles became apparent. In conclusion, silencing of the *BMP4* gene using transplacental RNAi injection can induce formation of colorectal polyps in mice.

## Introduction

Colorectal cancer has a high incidence and is associated with high mortality worldwide (1–6). In 2011, colon cancer ranked third among emerging cancer types and second in cancer-related causes of death in the USA (1). A high incidence of colon cancer (79/100,000) was also reported in Japan (2). The incidence of colorectal neoplasms is associated with the environment, gender and ethnicity (3). Their malignant transformation rate is associated with the pathological status of the neoplasm. Carcinogenesis occurs in nearly 100% of familial polyposis cases. The 5- and 10-year carcinogenesis rates of adenomatous polyposis are 4 and 14%, respectively (4). In colorectal carcinoma patients <18 years, the overall survival is 23% at 5 years (5). Colorectal cancer has a progression model of polyps-adenoma-adenocarcinoma, and most intestinal neoplasms are manifestations of the precancerous pathological stage of colorectal cancer (1,6).

The etiology of colorectal cancer is unclear, and early diagnosis is difficult (7). Elucidation of the mechanisms of oncogenesis in colorectal cancer mainly relies on animal models (8). Animal models of colorectal cancer can be established using skin injection of xylene (9), subcutaneous injection of primary cells in nude mice (10), primary cell implantation (11), high-fat, high-sugar diet (12) and *APC* gene knockout in mice (13,14).

The bone morphogenetic protein 4 (BMP4) is a member of the transforming growth factor β (TGF-β) superfamily, and regulates the early embryonic development of organs and systems, including bones (15), the nervous (16), digestive (17) and circulatory system (18). Adenomatous polyposis coli (*APC*) is a colon tumor suppressor gene. In juvenile polyposis, changes in the *BMP4* gene may precede *APC* mutations (19–21). However, similar studies found numerous additional molecular events occurring prior to *APC* mutations (13,14). However, no prospective study has been reported to date (22).

Inhibition of the noggin-BMP-Smad signaling pathway can induce colorectal polyps in mice (23). RNA interference (RNAi) is a method to silence gene expression, reported to have dose-dependent effects (24). This method can be used to reduce gene expression in the offspring by injection of different doses of endo-free plasmid, or by adjusting the intervention time at pregnancy (25,26). Gene silencing by RNAi has unique advantages in the study of developmental disorders (27–29). Methods for transplacental injection of RNAi were first described by O’shea *et al* in 2006 (26). The RNAi effects were apparent from 6.5 days post coidum (dpc) and disappeared at 17.5 dpc (26).

The present study provides a new approach for studying the molecular events occurring in intestinal polyps. In order to provide a rapid and stable model of colorectal polyps, we constructed an F1 mouse colorectal polyp model using transplacental RNAi technology to silence the *BMP4* gene. Growth and development of intestinal polyps were observed following *BMP4* silencing. The expression of BMP4 was monitored using western blot analysis and reverse transcription-polymerase chain reaction (RT-PCR) and that of *Smad4* was also studied using RT-PCR. Our study contributes in the understanding of the mechanisms underlying colorectal disease.

## Materials and methods

### Animals and ethics

Balb/c mice, 8–12 week-old and weighing 14–18 g, were purchased from the Animal Center of the Monash University, Australia (n=8) and the Animal Center of Chongqing Medical University, China (n=56). The experiments were performed using protocols approved by the Institutional Animal Care and Ethics Committee (IAC-EC) at the Chongqing Medical University (ethics certificate no. 20120019). Mice were kept in a specific pathogen-free facility room at the Chongqing Medical University Animal Center, with temperature at 25±2°C, 50±5% humidity, and a 12-h light/12-h dark cycle; water and food were provided *ad libitum*. Male and female mice were kept in the same cages at a 2:1 ratio. A female mouse in which a vaginal plug was found in the next morning was marked as 0.5 dpc and kept in a single cage. The offspring mice were sacrificed by cervical dislocation at 1, 4 and 8 weeks following birth. Disposition of the animals at the end of study, and sacrifice criteria and methods were all in accordance with the Code of Practice for the Care and Use of Animals for Scientific Purposes (IAC-EC).

### Experimental groups

Pregnant mice were randomly divided into three groups: Ringer’s, pSES and pSES-BMP4, injected with 10 μl/g Ringer’s solution provided by Chongqing Children’s Hospital, 50 μg/μl pSES empty vector, and 50 μg/μl pSES-SiBMP4 vector, respectively. The pSES vectors bear a copy of the entire DsRed coding region, allowing fluorescent detection of the delivered plasmids. Injections were performed from the tail vein at 9.5 dp, with a volume of 10 μl/g (if a mouse is 20 g, the volume is 200 μl). The injection time was 5 sec ±10%. Plasmids for siRNA were purchased from the Oncogene Laboratory, Biological Sciences Division, University of Chicago (Chicago, IL, USA), and contained the following 4 sites of RNAi to silence the BMP4 gene: aGGTCCAGGAAGAAGAATAAtttt; aACGAAGAACATCTGGAGAAtttt; aGAGCCATGCTAGTTTGATAtttt; aGGGAAAAGCAACCCAATTAtttt. The F1 offspring from the pSES-BMP4 group (colorectal cancer model) were analyzed by RT-PCR and western blotting at 1, 4 and 8 weeks after birth. The F1 mice were sacrificed by cervical dislocation, then the intestinal tissue was removed and rinsed in phosphate-buffered saline at 4°C. Part of the intestinal tissue samples was stored at −80°C and used for intestinal DsRed fluorescence observations as described in the following section. The remaining samples were stored in 4% paraformaldehyde and used to examine the morphology of the area from the ileocecal junction to the anus intestine under a stereomicroscope (SMZ1500; Nikon, Tokyo, Japan).

### Fluorescence microscopy

The frozen intestinal sections were excited with a green laser (Nikon AIR; Nikon, Tokyo, Japan), and DsRed fluorescence was detected using a Nikon A1R laser scanning confocal microscope. The Image-Pro Plus 6.0 image analysis system (Media Cybernetics Inc., Rockville, MD, USA) was used to quantify the fluorescence intensity values.

### Hematoxylin and eosin (H&E) staining

Tissues were immediately fixed in 4% buffered formalin for 48 h, embedded in paraffin, and sectioned at 5 μm thickness. We observed the tissues using the Nikon Eclipse 55i microscope.

### RT-PCR detection of BMP4 and Smad4 genes

Total RNA was extracted from tissues with the TRIzol reagent (Invitrogen Life Technologies, Carlsbad, CA, USA) according to the manufacturer’s instructions. To generate cDNA, reverse transcription was carried out with the Prime Script RT Enzyme mix reverse transcriptase. Then, the cDNA samples were amplified by PCR using the following cycling conditions: 94°C for 5 min, followed by 39 cycles at 94°C for 30 sec; annealing temperature (see below, primers) for 30 sec; 72°C for 30 sec, followed by a final step at 72°C for 5 min. Oligonucleotide primers, purchased from Invitrogen Life Technologies, and the corresponding annealing temperatures (At) for amplification of each gene were the following: i) bone morphogenetic protein 4 (BMP4) forward (F), GACTTCGAGGCGACACTTCT; reverse (R), CCTGGGATGTTCTCCAGATG; At, 60°C, ii) Smad4 F, CATTCCAGCCTCCCATTTCCAATC; R, CACATAGCCATCCACAGTCACAAC; At, 55°C, iii) glyceraldehyde 3-phosphate dehydrogenase (GAPDH) F, AGGCCGGTGCTGAGTATGTC; R, TGCCTGCTTCACCACCTTCT; At, 58°C, iv) β-actin F, AAGATGACCCAGATCATGTTTGAGACC; R, GCCAGGTCCAGACGCAGGAT; At, 56°C. The amplified products were resolved on ethidium bromide-stained 2% agarose gels. Their densities were analyzed using the Quantity One software (Bio-Rad Laboratories, Inc., Hercules, CA, USA). Gene expression of target genes was expressed relative to that of the control (housekeeping gene) *β-actin*.

### Western blotting analysis of BMP4

Protein extracts were prepared from intestinal tissues (colon, cecum and rectum). The tissue samples were homogenized in RIPA lysis buffer and phenylmethanesulfonyl fluoride (both from Beyotime Institute of Biotechnology, Shanghai, China) and proteins were directly extracted according to the manufacturer’s instructions of the protein extraction reagent. Protein concentrations were determined using a Micro Bicinchoninic Acid (BCA) Protein Assay reagent (Beyotime Institute of Biotechnology). Protein samples were then diluted to obtain equal (50 μg) protein amounts and heated at 100°C in an equal volume of sodium dodecyl sulfate (SDS) loading buffer (Beyotime Institute of Biotechnology) for 10 min. Proteins were then separated by SDS-polyacrylamide gel electrophoresis (5% spacer gel, 40 V, 50 min; 8% separating gel, 80 V, 70 min). Proteins were then electrophoretically transferred (Bio-Rad Laboratories, Inc.) to polyvinylidene fluoride membranes (Millipore Corp., Billerica, MA, USA) for 1.5 h at 250 mA. To block non-specific binding, the membranes were incubated with 5% bovine serum albumin in Tris-buffered saline with Tween 20 (TBST) at 37°C for 1.5 h. The membranes were then incubated overnight at 4°C with rabbit anti-BMP4 primary polyclonal antibody (1:400; Abcam, Cambridge, MA, USA). Next, the membranes were incubated with peroxidase-conjugated secondary anti-rabbit IgG (1:4,000; Zsbio, Beijing, China) according to the manufacturer’s instructions. The protein of interest was visualized using an enhanced chemiluminescence (ECL) western blotting substrate (Beyotime Institute of Biotechnology) and its relative expression was quantified using the Chemidoc XRS gel imaging system (Bio-Rad Laboratories, Inc.).

### Statistical analysis

All values were expressed as mean ± standard deviation (SD). The differences among groups were analyzed by a one-way ANOVA and t-tests implemented in the SPSS 17.0 software (SPSS, Inc., Chicago, IL, USA), followed by Student-Newman-Keuls (NKS)-q tests. P<0.05 were considered to indicate statistically significant differences.

## Results

### BMP4 and Smad4 are silenced in the intestines of F1 mice

**Figure 1:**
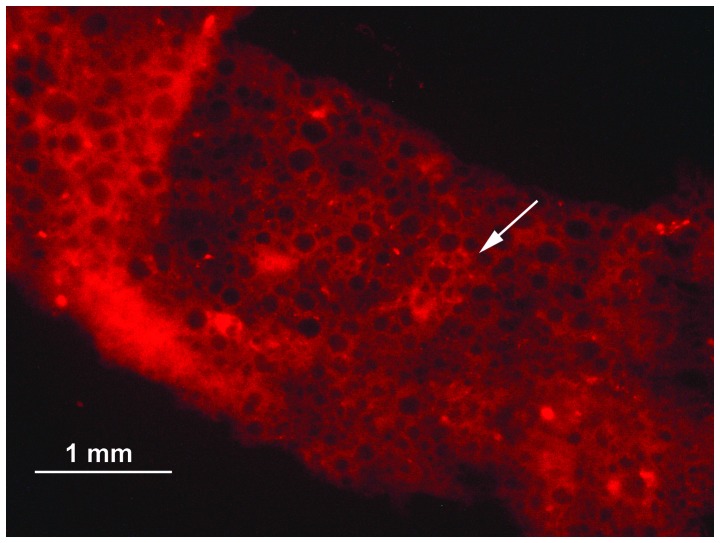
Transplacental BMP4 detection via DsRed fluorescence in the mouse colorectal cancer model at 1 week (magnification, ×40).

Following transplacental injection of the RNAi vector pSES-SiBMP4, the intestine showed circular red fluorescence, and the cytoplasm and nuclei of the intestinal gland cells showed red punctate fluorescence, indicating successful plasmid injection (Fig. 1).

**Figure 2:**
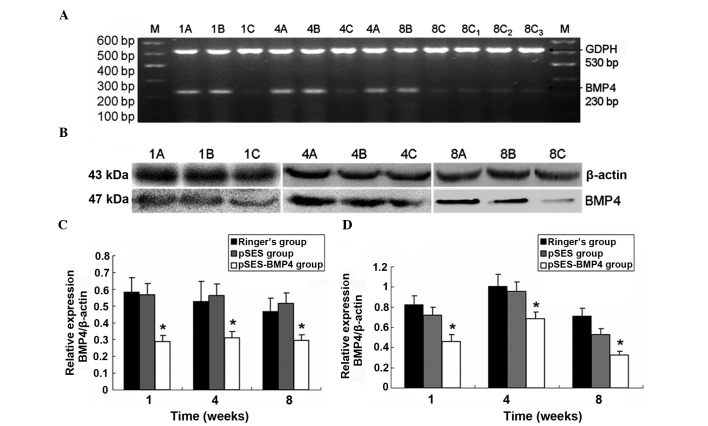
Expression of bone morphogenetic protein 4 (BMP4) at the gene and protein level in the mouse colorectal cancer model and two control groups. (A) Image of a gel with reverse transcription-polymerase chain reaction (RT-PCR) products, (B) western blot gel image, (C) western blotting analysis, and (D) RT-PCR analysis results (relative expression levels). 1, 1 week; 4, 4 weeks; 8, 8 weeks; A, Ringer’s group; B, pSES group; C, pSES-BMP4 group; C1, ileocecal colon; C2, transverse colon; C3, rectum; and M, DNA marker. ^*^P<0.05 compared to the pSES and Ringer’s groups.

**Figure 3:**
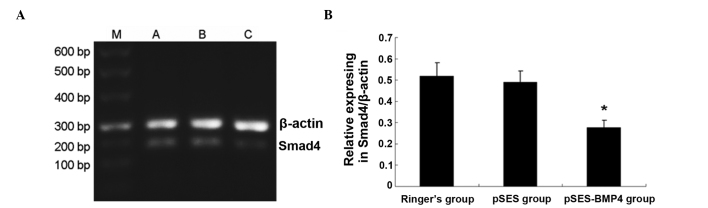
Expression of the *SMAD4* gene in the mouse colorectal cancer model and the two control groups. (A) Image of a gel with reverse transcription-polymerase chain reaction (RT-PCR) products, and (B) RT-PCR analysis results (relative expression levels). A, Ringer’s group; B, pSES group; C, pSES-BMP4 group; M, DNA marker. ^*^P<0.05 compared to the pSES and Ringer’s groups.

The relative mRNA level of the *BMP4* gene in the intestine of F1 mice was significantly reduced (P<0.05) in the pSES-BMP4 group compared to the Ringer’s and pSES groups at 1, 4 and 8 weeks after birth (Fig. 2A and C). In the 8-week-old F1 mice of the pSES-BMP4 group, the rectal *BMP4* level (0.11±0.01) was significantly reduced (P<0.05) compared to that of the transverse (0.15±0.01) and ileocecal colon (0.15±0.01) (Fig. 2A). The expression of the *Smad4* gene did not significantly change at 1 and 4 weeks, but significant differences were found at 8 weeks, when the *Smad4* mRNA level was significantly (P<0.05) reduced (Fig. 3).

Western blotting results showed that the intestinal BMP4 protein level in the pSES-BMP4 group was significantly reduced compared to that of the Ringer’s group at the same time-point (P<0.05). The intestinal BMP4 level in F1 mice of the pSES-BMP4 group was reduced by 46, 56, and 26% at 1, 4 and 8 weeks, respectively, compared to the Ringer’s group (Fig. 2B and D).

### Formation of colorectal polyps in F1 mice induced by transplacental BMP4 RNA interference (RNAi)

**Figure 4:**
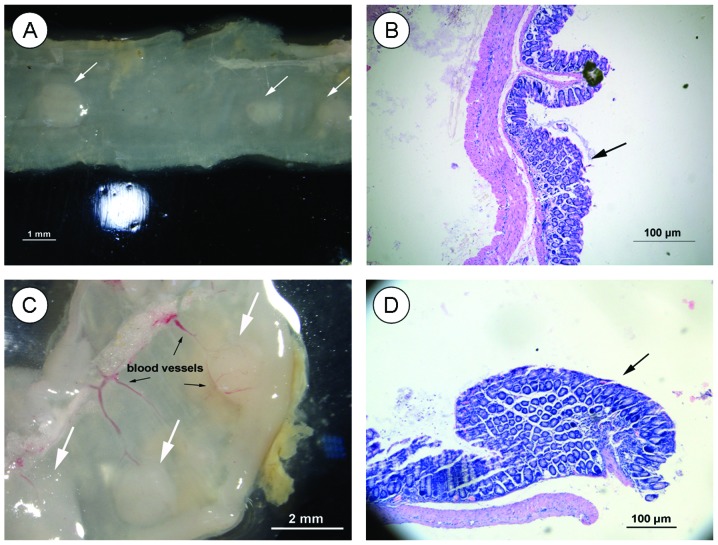
Histological examination of the mouse colorectal cancer model in the pSES-BMP4 group at (A) four weeks, intestinal mucosa (magnification, ×10), (B) four weeks, haematoxylin and eosin (H&E) staining (magnification, ×40), (C) eight weeks, intestinal mucosa (magnification, ×10), and (D) eight weeks H&E staining (magnification, ×40). Arrows denote colorectal cancer.

In the pSES-BMP4 group, F1 mice showed rectal bleeding 4 days following birth. The number of mice showing rectal bleeding increased after 4 weeks. In F1 mice in the Ringer’s and pSES groups, rectal bleeding was not observed. At 1, 4 and 8 weeks, intestinal samples were collected from F1 mice of the 3 different groups. Intestinal developmental changes were most obvious in F1 mice of the pSES-BMP4 group. At 1 week, the structure of the intestinal wall of mice in the pSES-BMP4 group was very similar to that of the control group. The intestine was transparent and smooth, and normal muscle and glandular cells were visible under the microscope. More than 5 polyps were formed in all 4-week-old mice in the pSES-BMP4 group, the intestinal folds were interrupted, and dew-like polyps formed above the plane. In 21 out of 29 F1 mice (72%), polyps accumulated near the rectum, and a small number of polyps was observed at the transverse and descending colon. At 4 weeks, intestinal endoscopy in the pSES-BMP4 group showed 6–8 layers of protruding intestinal gland structures with irregular arrangement, and pathological classification results for these samples indicated that they are similar to human juvenile polyposis samples (Fig. 4A). Microscopic examination of the intestinal wall of control mice at 8 weeks revealed regular folds of the intestinal epithelium parallel to the long axis and 2–3 layers of gland cells. Colorectal polyps appeared in the intestinal wall of all 8-week-old mice in the pSES-BMP4 group; the polyps were increased significantly in size and showed a crisp texture. Thick branch vessels grew into the polyps and stiff blood vessels were visible on the surface, while pedicles were also formed (Fig. 4C). Under the microscope, 10–15 layers of irregularly arranged glands were observed parallel to the intestinal wall, and glandular polarity was abnormal (Fig. 4D). Blood vessels grew into the surface and the parenchyma, showing hamartoma-like growth (Fig. 4C). At 8 weeks, the intestinal folds of mice in the control groups were obvious, and 1–2 layers of glands perpendicular to the intestinal wall were visible under the microscope.

## Discussion

RNAi is a common technique to study gene function (27,30). In this study, we delivered an BMP4-RNAi plasmid in the colon of F1 mice, as confirmed by positive DsRed fluorescence in the colon. The results of RT-PCR and western blotting analyses showed that in F1 mice of the pSES-BMP4 group, the *BMP4* gene is successfully silenced. In addition, daily observations, including anatomical examinations, showed that these mice displayed rectal bleeding and formation of colorectal polyps. These results indicate that it is feasible to obtain an F1 generation with reduced expression of *BMP4* via transplacental RNAi injection in pregnant mice, and thus successfully establish a mouse model of colon polyps, which offers important advantages in studying the role of the *BMP4* gene in the occurrence and development of colorectal polyps and cancer.

In this study, a high number of offspring with reduced *BMP4* gene expression was obtained through silencing of the *BMP4* gene via transplacental RNAi injection of 9.5 dpc pregnant mice. The phenotypic changes of the mice were observed by microscopical and morphological examination of tissue sections, and H&E staining. Reduced expression of the target gene was verified at both the genomic and proteomic levels. The incidence of colorectal polyps in the pSES-BMP4 group at 8 weeks (100%) was higher than at 4 weeks (72%). Hematochezia, as well as multiple polyps (>5) of considerable size and irregular shapes were observed. The arrangement of the glands was altered when observed under the microscope, and the polyps showed a tendency to become malignant. In addition, rectal polyps accounted for 72% of all polyps. The distribution of colorectal polyps in mice was consistent with the polyp distribution observed in the clinic, and the mRNA level of the *BMP4* gene was significantly reduced in the rectum compared to the colon, which might explain why rectal polyps are more common in the clinic. The above results showed that transplacental RNAi injection allows silencing of the *BMP4* gene in order to establish an F1 mouse model of colorectal polyps. Results of the present study laid a theoretical foundation for clarifying the mechanism of colorectal polyps and carcinogenesis.

In summary, this study successfully established a stable F1 mouse model of colorectal polyps through transplacental RNAi injection. The advantage of this technique is that it allows silencing gene expression in mice rapidly (within 1 month) and cost-effectively. A high number of F1 mice with highly similar genes can be obtained, which is of great importance for monitoring the phenotype and genotype of F1 mice and studying the occurrence and development of genetic diseases. However, 60% of pregnant mice in the pSES-BMP4 group experienced incomplete abortion. The natural life span of mice with the silenced *BMP4* gene was up to 13 weeks, which is longer than that of mice where *BMP4* RNAi was introduced at the late cleavage-stage of embryonic development (31). F1 mice may have combined nervous and/or circulatory system abnormalities, but the incidence of these abnormalities is reduced compared to *BMP4* knockout mice (29,32).
